# Trends in spatio-temporal dynamics of visceral leishmaniasis cases in a highly-endemic focus of Bihar, India: an investigation based on GIS tools

**DOI:** 10.1186/s13071-018-2707-x

**Published:** 2018-04-02

**Authors:** Rakesh Mandal, Shreekant Kesari, Vijay Kumar, Pradeep Das

**Affiliations:** Department of Vector Biology and Control, Rajendra Memorial Research Institute of Medical Sciences (ICMR), Agamkuan, Patna, Bihar 800 007 India

**Keywords:** Kala-azar, Spatio-temporal analysis, Spatial autocorrelation, Geographical Information Systems

## Abstract

**Background:**

Visceral leishmaniasis (VL) in Bihar State (India) continues to be endemic, despite the existence of effective treatment and a vector control program to control disease morbidity. A clear understanding of spatio-temporal distribution of VL may improve surveillance and control implementation. This study explored the trends in spatio-temporal dynamics of VL endemicity at a meso-scale level in Vaishali District, based on geographical information systems (GIS) tools and spatial statistical analysis.

**Methods:**

A GIS database was used to integrate the VL case data from the study area between 2009 and 2014. All cases were spatially linked at a meso-scale level. Geospatial techniques, such as GIS-layer overlaying and mapping, were employed to visualize and detect the spatio-temporal patterns of a VL endemic outbreak across the district. The spatial statistic Moran’s I Index (Moran’s I) was used to simultaneously evaluate spatial-correlation between endemic villages and the spatial distribution patterns based on both the village location and the case incidence rate (CIR). Descriptive statistics such as mean, standard error, confidence intervals and percentages were used to summarize the VL case data.

**Results:**

There were 624 endemic villages with 2719 (average 906 cases/year) VL cases during 2012–2014. The Moran’s I revealed a cluster pattern (*P* < 0.05) of CIR distribution at the meso-scale level. On average, 68 villages were newly-endemic each year. Of which 93.1% of villages’ endemicity were found to have occurred on the peripheries of the previous year endemic villages. The mean CIR of the endemic villages that were peripheral to the following year newly-endemic villages, compared to all endemic villages of the same year, was higher (*P* < 0.05).

**Conclusion:**

The results show that the VL endemicity of new villages tends to occur on the periphery of villages endemic in the previous year. High-CIR plays a major role in the spatial dispersion of the VL cases between non-endemic and endemic villages. This information can help achieve VL elimination throughout the Indian subcontinent by improving vector control design and implementation in highly-endemic district.

## Background

Visceral leishmaniasis (VL), or kala-azar, is a vector-borne neglected tropical disease that is a serious public health concern in India. VL is anthroponotic caused by the parasite *Leishmania donovani* and is spread to humans by the sand fly *Phlebotomus argentipes* (Diptera: Psychodidae) in the Indian subcontinent (ISC) [1, 2]. VL is endemic in more than 80 countries around the globe [3, 4] and there are an estimated 0.2–0.4 million cases and 20,000–40,000 deaths each year [5]. Over 90% of new cases globally occur in only six countries: Brazil, Ethiopia, India, Somalia, South Sudan and Sudan [4]. India alone reports more than 80% of ISC cases annually [6].

In India, VL is endemic in the 55 districts of four middle-eastern states: Bihar, Jharkhand, Uttar Pradesh and West Bengal. In these states, an estimated 130 million are at risk. Bihar is the worst affected state, contributing more than 70% of Indian cases annually [7]. The three strategies (early case detection, effective treatment and vector control) are the main pillars in achieving the elimination target (i.e. less than 1 case/10,000 at sub-district/block level) [3, 4]. Indoor residual spraying (IRS) using dichlorodiphenyltrichloroethane (DDT) at 1 g/m^2^ is the only method for sand fly vector control in India since 1977 [3]. However, in 2015, DDT was replaced by synthetic pyrethroid (alphacypermethrin) due to widespread resistance development in sand flies, including *P. argentipes* [7–10]. In a few places, IRS is combined with environmental manipulation to reduce the population density at breeding and resting sites [11].

However, despite these promising efforts, VL cases still arise in the poorest settings of these endemic districts (458 blocks in 34 districts) of Bihar and gradually spread to new areas. The number of endemic districts increased from 28 in 1977, to 31 in 2007, 33 in 2011 and 34 in 2015 [12–14]. Implementation of the vector control interventions are based on passive reporting of VL cases in the last 3 years, including the implementation year [6, 10]. It is still unknowing how to select the villages (potential for VL transmission) for targeted control interventions. Additionally, research on the trends in spatio-temporal dynamics of VL cases is not yet fully explored. Therefore, it is important to understand the spatio-temporal dynamics of VL transmission at a meso-scale level. Geographical information systems (GIS) are an integrated set of tools that allow both the analytical manipulation and the visual presentation of public-health events by accounting for space and time [15–17]. GIS links both non-geographical and geographically referenced data with graphical map features to allow a broad range of geospatial analyses as well as map production [18]. Epidemiological studies have used GIS techniques for disease mapping, visualization and cluster detection [19–21]. The range of map visualizations helps scientists, researchers and public health personnel to communicate this complex information to the public and policy makers simply.

An improved understanding in spatio-temporal distribution of cases may improve surveillance and control strategy implementation. This study explores the trends in spatio-temporal dynamics of VL endemicity between the villages in Vaishali District of Bihar State using GIS tools and spatial statistical analysis.

## Methods

### Study area description

**Fig. 1:**
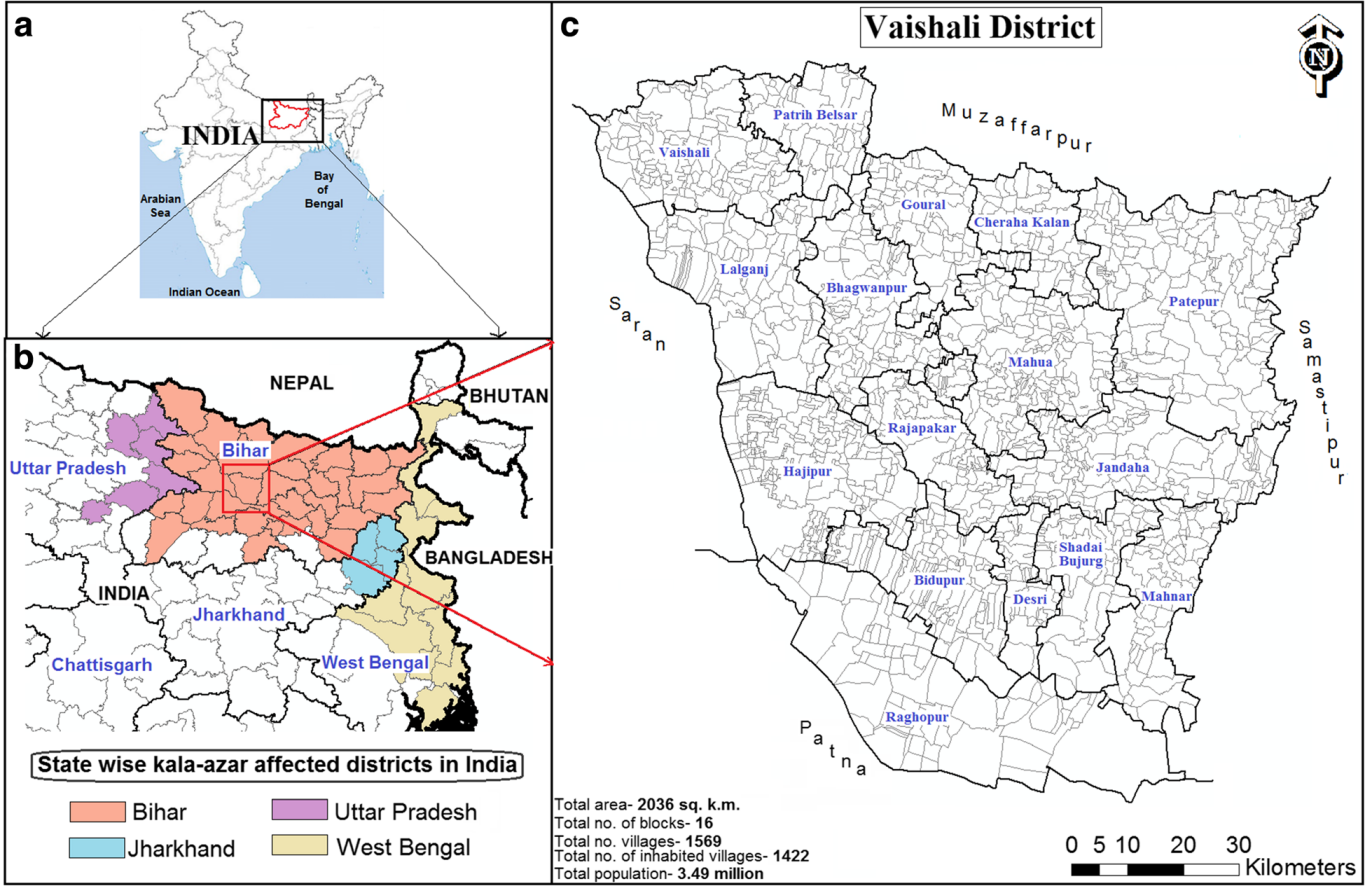
Map of the study area. **a** The location of Bihar State in India. **b** The location of Vaishali District among the other VL endemic districts in Bihar. **c** The spatial distribution of the 16 Vaishali blocks and the villages boundary within them

We conducted this study in the villages of Vaishali District of Bihar State, India, where VL has been highly-endemic for several decades. Vaishali lies between latitudes 25°28'–26°05'N and longitudes 85°05'–85°40'E and shares boundaries with four other highly-endemic districts: Muzaffarpur in the north, Patna in the south, Samastipur in the east and Saran in the west (Fig. 1). Vaishali covers a total area of 2036 km^2^ with a population of 3.49 million people (1717 people/km^2^). There are 1569 villages (1422 inhabited and 147 uninhabited) distributed over 290 Gram panchayats (GPs) in the 16 Community Development (CD) blocks.

### Epidemiological and population data collection and analysis

We obtained individual-level annual data on VL cases from the vector-borne disease control office (VBDCO, Hajipur) of Vaishali District from 2009 to 2014. The data include information on name, gender, age, current-address (including block, GP, village and hamlet names) and VL diagnosis date. We divided the annual datasets into two groups for analysis. Each group contained 3 years of cases, in line with IRS-based VL vector control program conducts in Bihar, India [6, 10]. The VL endemic data (villages and cases) between 2012 and 2014 were used in the spatial analysis and VL cases between 2009 and 2011 were used to inform the endemic history of the study villages; an endemic village is defined as one or more cases in a calendar year. Newly-endemic (no case in the past 3 years of the study year) and pre-endemic (one or more cases at any point in time during the past 3 years of the study year) villages were identified based on the VL endemic history of the study villages in the last 3 years.

We used population data from the 2011 Indian census to estimate the villages’ population from 2012 onwards, based on a mean district wise annual growth rate of 2.5% [22]. The annual case incidence rate (CIR; 2011–2014) per 1000 people per village is calculated using the formula described by Indrayan et al. [23].

### Geodatabase development and epidemiological linking

We developed a digital geodatabase of GIS layers (as a shape file) including district, block and village administrative boundaries (at 1:5000 scale) using the block-cadastral map and the Survey of India’s (SOI) Toposheet map- 72G. For geodatabase-epidemiological linking, we performed a layer update for adding attribute information (such as revenue name, GP, block, police station and postal code) to village polygons. The cases were obtained from passive reporting to the VBDCO and checked for duplicates. All VL cases data were then cross-linked to village polygon names (in a GIS layer) by unique identification codes to facilitate meso-scale level data storing, integration, mapping, visualization and spatio-temporal analyses. We rectified post database errors (e.g. patient’s name and address) through field verification. All GIS analyses were carried out in ArcGIS v9.3 (ESRI, Redlands, CA, USA).

### Spatial-statistical analysis

Moran’s I Index, a spatial-correlation statistic, was used to explore the spatial pattern of CIR between endemic villages (using village political boundary) [24]. Moran’s I Index varies between +1 to -1; a positive value (> 0) indicates presence of clustering (either high-CIR village near high-CIR village or low-CIR village near low-CIR village), while a negative value (< 0) indicates dissimilar or variable pattern (either high-CIR village near low-CIR village or low-CIR village near high-CIR village). Moran’s I value of an absolute magnitude less than 0.3 suggests clustering (or, dispersion) probably occurring in a few regions; a value near 0 indicates an absence of spatial-correlation or a random pattern [25]. We used the ‘Z’ statistic to assess the significance of Moran’s I Index; an absolute score larger than 1.96 coincides with a significance level at *P* = 0.05 and was interpreted as significant [26].

### Statistical analyses

We used descriptive statistics such as mean, standard error (SE), confidence intervals (CI) and percentages to summarize the VL. A two-sample Student’s t-test was performed to determine the case occurrence difference between the years 2012–2014. All statistical analyses were performed using the SPSS software, v22 (SPSS Inc., Chicago, IL, USA).

## Results

### Descriptive analysis of VL cases at the meso-scale level

There were 2719 cases (average 906 cases/year) reported during 2012–2014, of which 2355 (86.6%) cases were reported from the pre-endemic villages and 362 (13.3%) from the newly-endemic villages. Table 1 shows the annual VL cases and incidences by newly- and pre- endemic villages. The CIR in the pre-endemic villages was higher (*t*_(616)_ = 2.06, *P* = 0.039) than the newly-endemic villages, in all the 3 years.

**Table 1:** Descriptive statistics of VL cases and CIR (per 1000 population) computed for the newly-endemic and pre-endemic villages of Vaishali District (Bihar) during 2012–2014

Year	Newly-endemic villages		Pre-endemic villages	
	95% CI of VL cases (mean ± SE)	95% CI of CIR (mean ± SE)	95% CI of VL cases (mean ± SE)	95% CI of CIR (mean ± SE)
2012	1–9 (2.14 **±** 0.19)	0.23–16.47 (2.28 **±** 0.37)	1–38 (3.71 **±** 0.33)	0.11–43.37 (2.44 **±** 0.31)
2013	1–4 (1.46 **±** 0.10)	0.12–10.42 (1.56 **±** 0.26)	1–21 (2.78 **±** 0.19)	0.09–32.57 (2.07 **±** 0.26)
2014	1–5 (1.61 **±** 0.12)	0.20–13.11 (1.19 **±** 0.20)	1–14 (2.56 **±** 0.18)	0.09–44.23 (1.77 **±** 0.29)
Total	1–9 (1.76 **±** 0.09)	0.12–16.47 (1.69 **±** 0.17)	1–38 (3.06 **±** 0.15)	0.09–44.23 (2.12 **±** 0.17)

### Spatio-temporal analysis of village’s VL endemicity

**Fig. 2:**
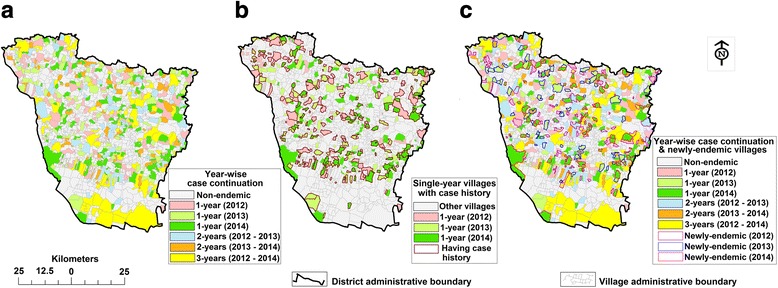
**a** Continuation of VL endemicity for study villages between 2012 and 2014. **b** Spatio-temporal distribution of study villages endemic for a single year (during 2012–2014) and non-endemic for one or two year within the last three years. **c** Spatio-temporal distribution of newly-endemic study villages (had no cases in the last three years) during 2012–2014

**Fig. 3:**
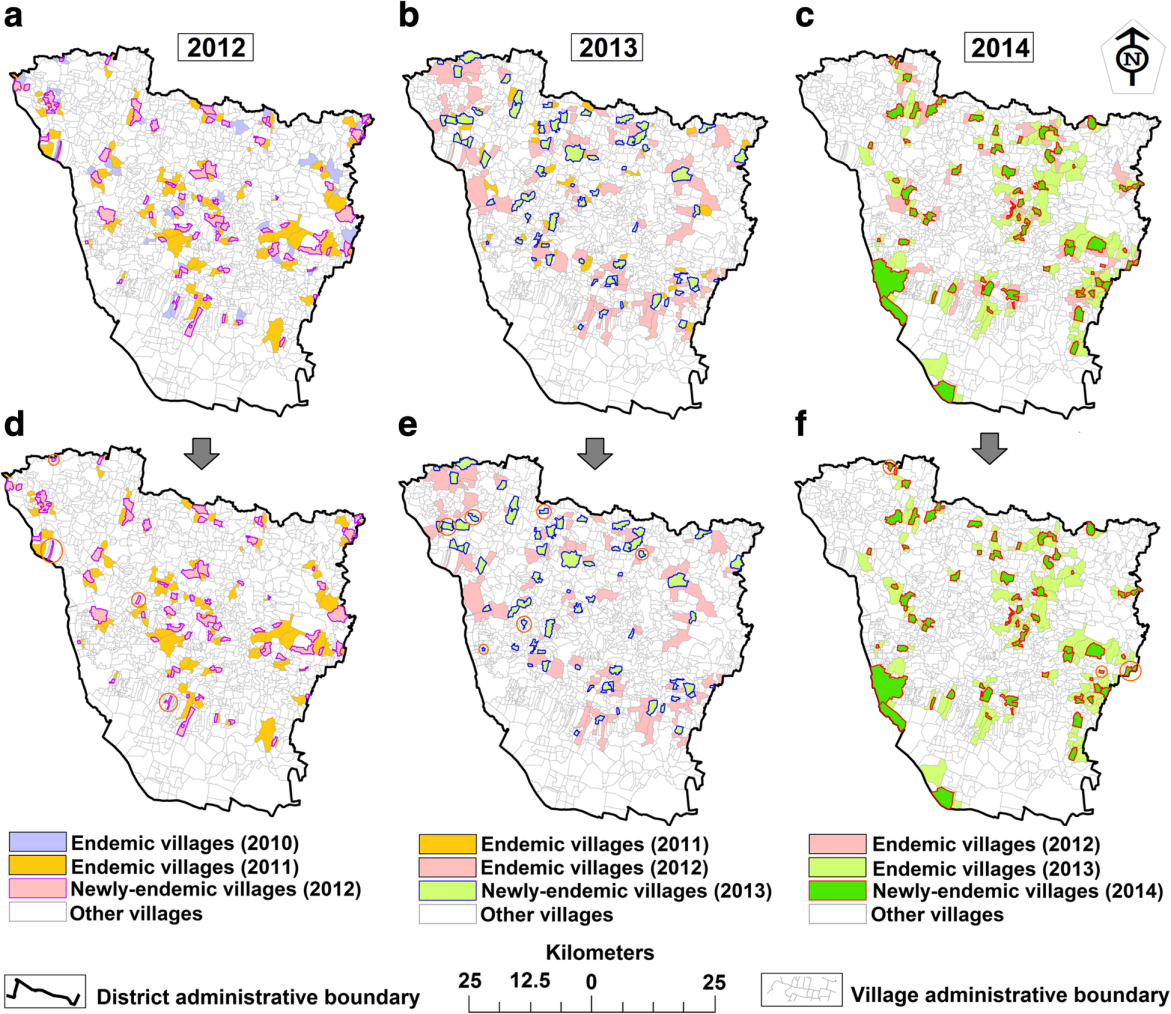
The newly-endemic and their peripheral pre-endemic villages, distributed across Vaishali District, Bihar (India), 2012–2014. **a**, **b** and **c** show the last two years of pre-endemic villages adjacent to the boundaries of newly-endemic villages during 2012–2014. **d**, **e** and **f** show the last one year of pre-endemic villages adjacent to the boundary of newly-endemic villages during 2012–2014. The newly-endemic villages with no peripheral endemic villages in the previous year marked by a solid brown line circle (**d**, **e** and **f** for 2012, 2013 and 2014, respectively)

We observed that 624 villages (43.9% of all inhabited villages) were endemic during 2012–2014; of which 232 (37.2%) remained endemic for more than 1 year, of which 154 (66.4%) for 2 years (56.5% of 2012–2013; 43.5% of 2013–2014) and 78 (33.6%) for 3 years (2012–2014) (Fig. 2a, Table 2). The remaining 392 (62.8%) villages were endemic for only a year; among these 223 (56.9%) villages were not endemic for periods of 1–2 years within the last 3 years (Fig. 2b). During the study period, a total of 204 (32.7%) villages were newly-endemic, of which 35 (17.2%; 24 for 2012 and 11 for 2013) remained endemic for 1–2 years [2012 and 2014 (*n* = 5); 2012–2013 (*n* = 12); 2012–2014 (*n* = 7); and 2013–2014 (*n* = 11)] (Fig. 2c). An average of 311 villages (21.9% of all inhabited villages) were endemic each year, of which 68 villages (21.9%) were newly-endemic. The most (average 93.1%, 63 village/year) newly-endemic villages were found to be reported on the peripheries of the previous year’s boundary with the endemic village (Fig. 3).

**Table 2:** Number of endemic villages common between years and remain endemic for one year computed for each year in Vaishali District (Bihar) during 2012–2014

Year	Common endemic villages between years			Single-year endemic villages per year	Total
	2012–2013 (2 years)	2013–2014 (2 years)	2012–2014 (3 years)		
2012	87	–	78	156	321
2013		67		99	331
2014	–			137	282
Total	87	67	78	392	624

### VL cases spatio-temporal distribution, CIR and Moran’s I statistic

**Fig. 4:**
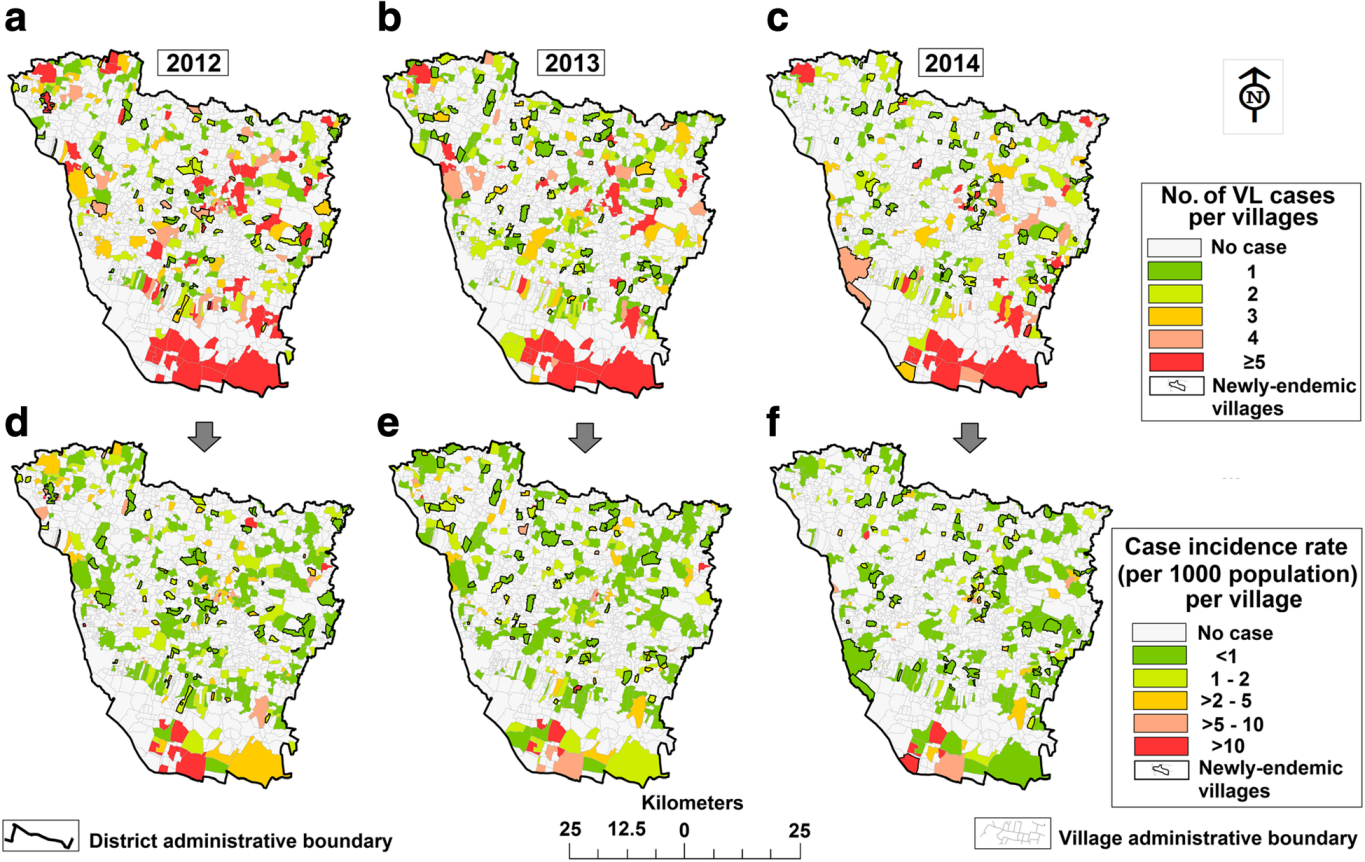
Spatial distribution of annual VL cases and CIR in Vaishali District, Bihar (India), 2012–2014 (**ad**, **be** and **cf.** for the years 2012, 2013 and 2014, respectively). The new village’s boundaries are marked by the solid black line

**Fig. 5:**
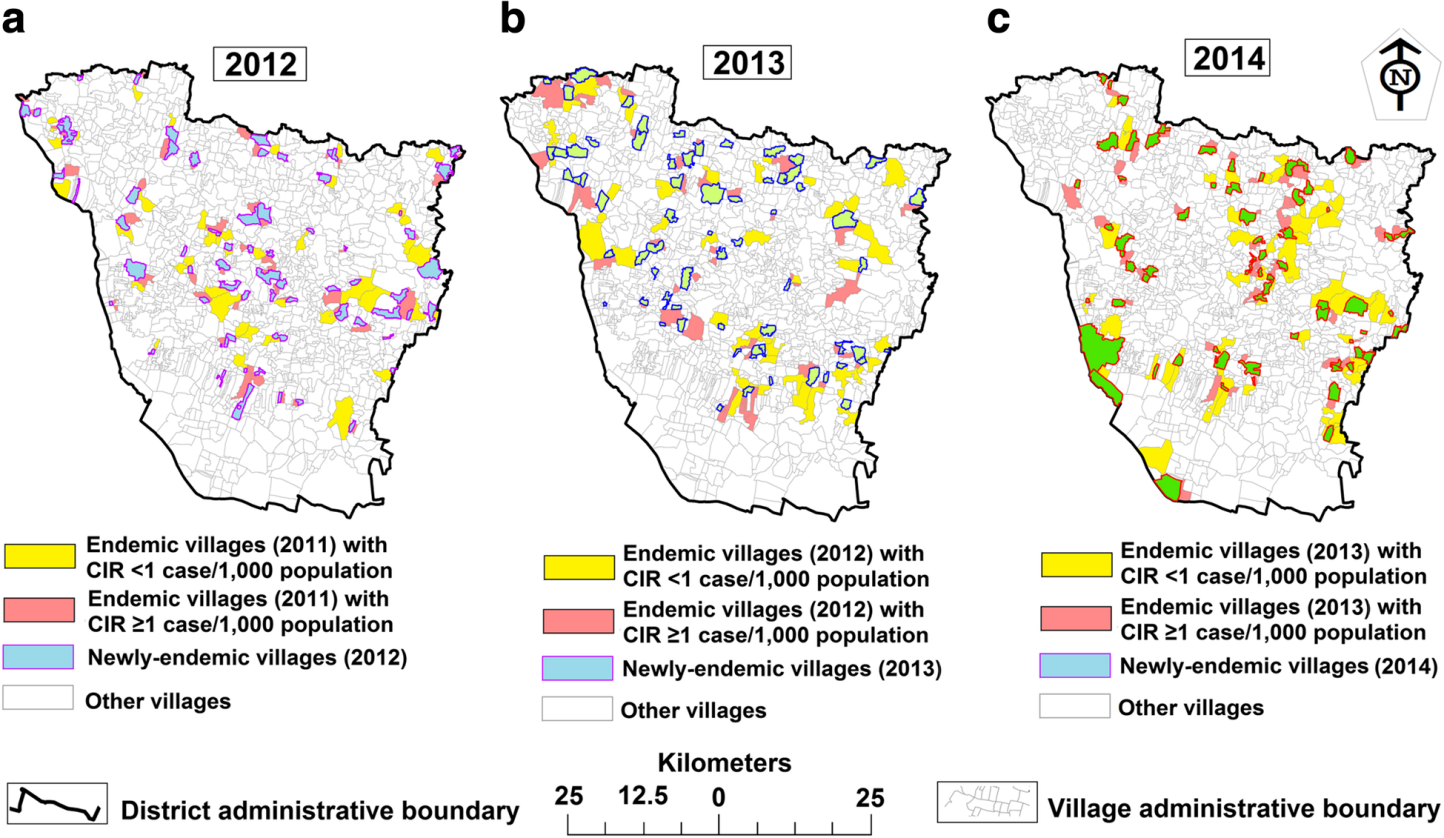
CIR for the endemic villages that were situated on the peripheries of following year’s newly-endemic villages: **a**, **b** and **c** for the years 2012, 2013 and 2014, respectively

The annual mean cases of endemic villages and their CIR were varied (*t*_(230)_ = 2.62, *P* = 0.009) between the study years (Fig. 4). An average of 138 endemic villages had an annual CIR (42.1% of all endemic villages) above one case/1000 population for all study years, of which 37.1% (mean: 48 village/year) of villages had a boundary adjacent to a newly-endemic village in the following year [covering 69.1% (mean: 47 village/year) of the total newly-endemic villages every year] (Figs. 4, 5). The overall mean CIR (during 2012–2014) of pre-endemic villages that were peripheral to the following year newly-endemic villages was 2.1 cases/1000 population [95% CI: 1.97–2.21], while all pre-endemic villages had an overall mean CIR of 1.83/1000 population (95% CI: 1.74–1.91). The mean values were varied significantly (at *P* < 0.05) in every study year. Table 3 summarizes the result of Moran’s I index statistic. Spatial distribution of CIR among the endemic villages was found to be clustered with positive Moran’s I (between 0.10 and 0.17) and significant (at *P* < 0.05).

**Table 3:** Spatial correlation measured by Moran’s I Index for the VL incidence rates among the endemic villages in Vaishali District (Bihar) during 2012–2014

Year	Moran’s I score	Z-score	*P*-value	Pattern evaluated
2012	0.17	2.46	0.01	clustering
2013	0.10	1.97	0.04	clustering
2014	0.11	2.74	0.01	clustering

## Discussion

In this study, we took advantage of recent advances in GIS tools to explore the spatio-temporal dynamics of VL endemicity in Vaishali District of Bihar State (India). Vaishali District reported a considerable number of VL cases (906 cases/year) along with a high coverage among villages (almost 44%) during 2012–2014; it is classified as a highly-endemic zone. The simultaneous occurrence of VL cases throughout the Vaishali District may have several causes, such as sand fly abundance throughout the year, presence of the parasite in the human host, cultural homogeneity among the low-income communities and other environmental risk factors (e.g. household type, climate, vegetation abundance and type) among the rural villages [27–30]. Our result showed that a large-number of VL cases had been reported from the pre-endemic villages with a high CIR (e.g. 2.1 cases/1000 population); among them, only few villages’ cases occurred at one- or two-year intervals. This finding suggests that in an endemic focus, an area with a previous history of VL may have a higher probability of future recrudescence. This could be due to the suboptimal implementation of IRS, delays between onset of VL symptoms and treatment and, in some places, insecticide resistance [8–10, 31, 32]. A similar result was obtained in a previous study conducted in Bangladesh [33]. Our results also showed that a few VL cases had occurred from the newly-endemic villages. Although newly-endemic villages are outnumbered almost five times compared to all endemic villages, a few newly-endemic villages had remained endemic for the next one or 2 years. In Bihar, the VL vector control program (using two rounds IRS of annually) is conducted in selected villages or hamlets which have been endemic for the last 3 years before the spray year [6, 10]. Only performing IRS in the endemic villages could explain the VL transmission to the newly-endemic villages or hamlets. Sand flies are targeted by IRS at their breeding and resting sites inside of human dwellings and cattle sheds, which cause them to move to another outdoor resting area (e.g. peri-domiciliary sparse-vegetation areas) and indoor sites (e.g. the nearest non-sprayed houses) [14, 34, 35]. Our results are in accordance with recent studies discussing an emergence of new ecological niches in previously non-endemic regions of India and Nepal [36, 37].

Although the VL cases had an uneven distribution over the study region, the endemic villages were found nearby each other. The meso-scale level case continuation mapping showed that the newly-endemic villages’ endemicity occurred on the peripheries of the endemic (last 2 years) village’s boundary and, interestingly, more than 93% of these newly-endemic villages were found to be reported on the peripheries of the previous year endemic villages. This result suggests that the presence of VL cases within the pre-endemic villages could increase the risk of VL infection in the adjacent non-endemic villages. Furthermore, the CIR showed that the pre-endemic villages that were peripheral to the following year newly-endemic villages had a higher mean compared to all endemic villages, in every study year. Although 42.1% of the endemic villages had an annual CIR above one case/1000 population, interestingly, a considerable number (69.1%) of the newly-endemic villages in the following year are found to be reported on the peripheries of these CIR-elevated villages. Thus, it is conceivable that previously-endemic villages with a high-CIR may increase the risk of VL case occurrence in the peripheral non-endemic villages. Moreover, the spatial-statistical analysis showed that the spatial distribution of CIR is non-random among the endemic villages and Moran’s I scores indicate clustering at the meso-scale level. This finding indicates that case occurrence among the endemic villages in a few pockets across the district had either high-CIR near high-CIR or low-CIR near low-CIR, confirming a previous finding [25].

The VL-epidemic pattern analyzed in this study is limited by underreporting, as we do not have the local case data for Vaishali District [38]. These underreported cases lead to an underestimate of the actual case number for the endemic villages and the ‘zero case’ villages. Nevertheless, this study executes the spatial-temporal analysis on the GIS-platform well to explore VL endemicity at the meso-scale level across Vaishali District; this cannot be understood through existing data or ground surveys.

## Conclusions

Our results indicate that there is a continuous, case dynamic interaction between endemic and non-endemic villages across the Vaishali District. The newly-endemic villages VL endemicity tended to occur on the peripheries of the previous year’s endemic villages boundary. An elevated CIR increased the risk of VL cases dispersal between endemic and non-endemic villages. Our study also demonstrated that the GIS tools and spatial statistics can be used as an epidemiological measuring tool to identify the risk and non-risk villages for VL transmission within a highly endemic region. These techniques not only provide an improved understanding of the distribution pattern of the disease, but also help to optimize the control resources more effectively. The results of this study may help public health scientists and researchers to design and implement control strategies in an advanced way to achieve the VL elimination target for the highly-endemic region of Bihar as well as others in the Indian subcontinent.

## Abbreviations

CD: Community development
CIR: Case incidence rate
DDT: Dichlorodiphenyltrichloroethane
GIS: Geographical information system
GP: Gram panchayat
IRS: Indoor residual spraying
VL: Visceral leishmaniasis
